# Clinical Remarks. Prof. Thomas F. Rochester, on Intermittent Fever, and on Pleurisy

**Published:** 1870-12

**Authors:** F. Bradnack

**Affiliations:** Member of the Class


### ART. IV.—*Clinical Remarks.* Prof. THOMAS F. ROCHESTER, *on Intermittent Fever, and on Pleurisy.*

REPORTED BY F. BRADNACK, MEMBER OF THE CLASS.

After speaking of the different forms of intermittent fever Prof. Rochester said:—If the disease appears to be malignant, it will be advisable to administer quinia, at any stage, rather than to wait for an interruption. Give for two or three days in doses of five grains, say twenty grains; next day give fifteen grains; next day ten grains; after this, for five days, give five grains per day. Other agents sometimes operate when quinia fails; of these, the best and most reliable is arsenic. All the so-called "patent" nostrums contain this metal. They are often combined with boneset, or some bitter drug, the maker desiring to carry the idea that the latter is the remedial agent. The dose of arsenic as an antiperiodic is from gr. $\frac{1}{20}$ to $\frac{1}{10}$, given three or four times in twenty-four hours. Fowler's solution is the best form in which to administer arsenic. The dose is from five to ten drops largely diluted, given after meals. It may be given alone, or in combination with quinia. Other remedies that have been used with more or less success in this disease, are sulphate of zinc, sulphate of copper, spider's-web, and various bitters. The remedy that is probably second-best to arsenic is common salt. This agent has undoubtedly valuable anti-periodic properties. To produce its effects, it should be given in the dry state. The great obstacle to its use consists in the fact that patients dislike to take it. Dr. Butler, of Saginaw, Mich., in a series of articles, published a few years ago in the *Buffalo Medical and Surgical Journal*, spoke very highly of its success in his hands, he having administered it to a large number of patients suffering from intermittent fever. It has been tried in this hospital (Sisters of Charity), given in doses of two or three drachms. It of course makes those who take it very thirsty. To allay this thirst, they drink, if allowed, largely of water, which, drinking very often, induces vomiting. I have never seen any serious gastric derangement follow the salt treatment. Before the war, Prof. William A. Hammond, of New York, wrote an article which attracted much attention, in which he strongly urged the claims of nitric acid to be considered as an anti-periodic, and doubtless it has valuable anti-periodic properties. There is an unfortunate prejudice in the minds of the public against the use of quinia, which prejudice has been much fostered by quacks, to the effect that this drug often produces permanent blindness and deafness in those who take it, leaving them the prey of worse ailments than those it was given to remedy. It need scarcely be remarked that this prejudice is foolish and unfounded. It would be hardly less unreasonable to accuse quinia of rendering men cripples or bankrupts. Doubtless, quinia may, in improper doses, occasionally produce unpleasant symptoms, and it has been known, when given carelessly, to produce deafness. To aver that these are its usual or frequent effects, is as absurd as it is incorrect. There is probably, on the other hand, no agent of equal potency, whose power for evil is so small. Of course, (as is the case with other drugs,) some persons are prevented from taking quinia by reason of an idiosyncrasy. Contrary to general opinion, small doses, say of one grain, are more apt to produce ringing in the ears than large ones, say of five grains. Experience demonstrates the truth of this statement. In consequence of intermittent fever the spleen is often greatly enlarged, which enlargement is not infrequently followed by dropsy. In these cases the administration of the tincture of the chloride of iron, (dose, grs. 10—15,) is often followed by great improvement. Sometimes quinia alone will suffice, for the removal of this condition. Common salt is said, by some observers, to reduce an enlarged spleen more rapidly than anything else. But, in this condition, the latest, and undoubtedly the best, remedy, is the bromide of potassium. Although, on their first appearance in the field of remedies, the bromides were probably over-estimated, still they are, doubtless, very valuable. In 1822 Dr. Toynbee, of Geneva, Switzerland, claimed that *bromine* exerted a remarkably curative influence on the enlarged spleen; but this agent, for various reasons, has never come into general use. The bromide of potassium, in large doses, will reduce an enlarged spleen very rapidly. It is generally imagined that the bromides and iodides produce (when given freely) anæmia and pallor of surface; but, in reality, the reverse of this proposition is generally true. They often improve the appetite.

Several months ago Mr. Hewson, in one of the London hospitals, under the service of Dr. Fuller, suggested the use of bromide of potassium in what is known as dumb ague, in cases which resisted the influence of quinia. Dr. Fuller found the effects of this remedy in these cases to be remarkable. In one case, a man who had taken ten grains of quinia per day, was put upon the use of forty grains of the bromide per day. In three days the paroxysms were interrupted, and in twenty grain doses per day continued for three weeks, the spleen, which had been greatly enlarged, was reduced to its normal size. His appearance improved. An increase in the number of red corpuscles occurred, and other effects of improved health were manifested. Dr. Fuller is a most reliable observer.

One of the patients in this hospital (Sisters of Charity) will, in lieu of more quinia for the cure of an attack of intermittent fever, be at once placed upon the use of bromide of potassium.

PLEURISY may be divided into two varieties, primary and secondary. Primary pleurisy comes under the head of diseases of idiopathic origin. The secondary form may arise from various causes, prominent among which are cold, blows or injuries, and other exciting causes, which may be briefly summed up under the term traumatic. Pleurisy, in its primary or idiopathic form, is probably a very rare disease. Double pleurisy occasionally occurs, but it is also rare. Louis says that, when double pleurisy exists, it is always an evidence of the simultaneous existence of tuberculosis, and it probably is dependent on tuberculous deposit. We often see cases which may be fairly denominated rheumatic pleurisy, for we find that the cause or causes which produce rheumatism also produce in these cases pleurisy.

In a case of the simplest form of uncomplicated pleurisy, the following symptoms are presented. The disease is probably ushered in by a well-marked chill, followed by febrile movement, accompanied by a lancinating pain in the vicinity of the nipple, which pain will generally travel through to the spinal column.

Inflammation in serous structures tends, as you are well aware, to travel over a greater part, or the entire surface of the membrane which it happens to attack. But in some instances the inflammation is so intense as to *circumscribe itself*—a lymph wall of circumvallation is thrown out,—in other words, the fire is so fierce that it puts itself out. Lymph may next be thrown out, which agglutinates the surfaces of the pleuræ. Acute pleurisy generally occurs suddenly. In the patient before us this morning we have probably a case of secondary pleurisy. One symptom, which would lead to this conclusion, is the great emaciation of the patient. It is also very probable that empyema also exists; there is certainly a large amount of fluid of some sort in the chest, the presence of which occasions much dyspnœa. Now, what in this case is the treatment indicated? Manifestly this is no case for depletion, neither would counter-irritation be of any avail; on the other hand, it would be apt to give rise to fever or to pustulation. Therefore, the treatment will be chiefly hygienic ; we shall administer diuretics, also diaphoretics if there be much fever. If fluid accumulates in the chest, it must be drawn off. We will not allow the patient to be suffocated by fluid in his own thorax. He is taking now five grains of carbonate of ammonia every three hours. This acts, in the first place, as a diffusible stimulant; secondly, as an expectorant; and it also frequently plays the part of a diaphoretic. It is, therefore, a valuable remedy in these cases. It is often very desirable to promote free expectoration in this disease, that the patient may be enabled to throw off much of the accumulated fluid in this way, if possible. This patient is also taking small doses of iodide of potassium thrice daily. Experiments have recently been made in one of the London hospitals of treating pleurisy patients with what may be defined as a dry diet—that is, they are fed, say, lamb chops and eggs, with just as little fluid as possible. The idea, of which this treatment is the exponent, is that when in any part of the system an abnormal amount of fluid has accumulated, nature will, if not hindered, draw off this surplus fluid to those parts of the economy from which it has been, as it were, fraudulently abstracted. This plan is said to work exceedingly well. This may be called the rational treatment. The great point in pleurisy is not to allow effusion to take place. It is often quite difficult to discover if the pleurisy is primary or secondary. If primary, and the patient be a person of robust constitution, the disease may be treated antiphlogistically with advantage. In such cases you have a remedy at hand which has too much gone into disuse—venesection. In the cases under consideration we are not allowed to omit it. If we do, we may be responsible for the patient carrying about a damaged chest the residue of his life. The present disuse of this potent remedy (either for good or evil) is an admirable illustration of the tendency of the human mind to run to extremes, for it would now seem impossible to assign any good reason for the abandonment of this remedy, except that in the past it was greatly abused. In the case of a robust individual, (as above supposed,) you may take with safety 12—20 or more ounces of blood, continuing its abstraction nearly to the point of syncope, then let the patient be put in bed and administer two grains of opium combined with a small quantity of ipecacuanha. Allow but little or no drink, and the disease will probably be found to be nipped in the bud.

